# Supplementary material for: β-Cyclodextrin-Based Poly (Vinyl Alcohol) Fibers for Sustained Release of Fragrances
Source: Polymers (Basel). 2022 May 13;14(10):2002. doi: 10.3390/polym14102002 (PMC9142904; doi:10.3390/polym14102002)
Supplement: Supplementary file 1 [file polymers-14-02002-s001.zip › polymers-1712901-supplementary.pdf]

β-Cyclodextrin based Poly (vinyl Alcohol) Fibers for Sustained Release of Fragrances

Chengyuan Xing [1,2], Xia Xu [1], Lei Song [1], Xiaoling Wang [1], Bangjing Li [2] and Kun Guo [1,*]

[1] College of Pharmacy, Southwest Minzu University, Chengdu 610041, China; xxia1011@163.com (X. X.); scsonglei@163.com (L. S.); wxl3232@sina.com (X. W.); guokun@swun.edu.cn (K. G.)

[2] Chengdu Institute of Biology, Chinese Academy of Sciences, Chengdu 610041, China; xing919969255@163.com (C. X.); libj@cib.ac.cn (B. L.)

* Correspondence: guokun@swun.edu.cn (K. G.); Tel.: +86-185-8339-6627

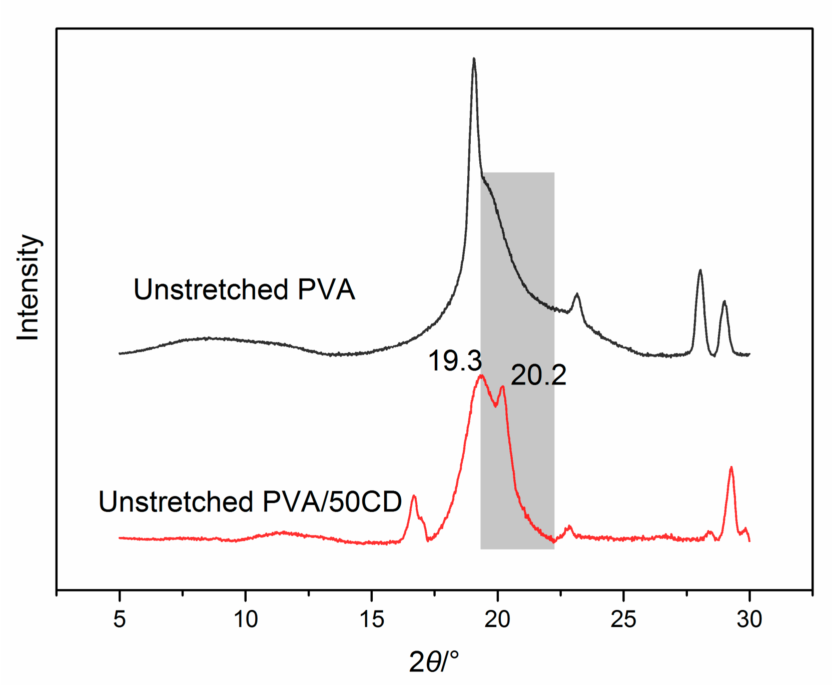

Figure S1. XRD of unstretched PVA and PCA/75CD fibers

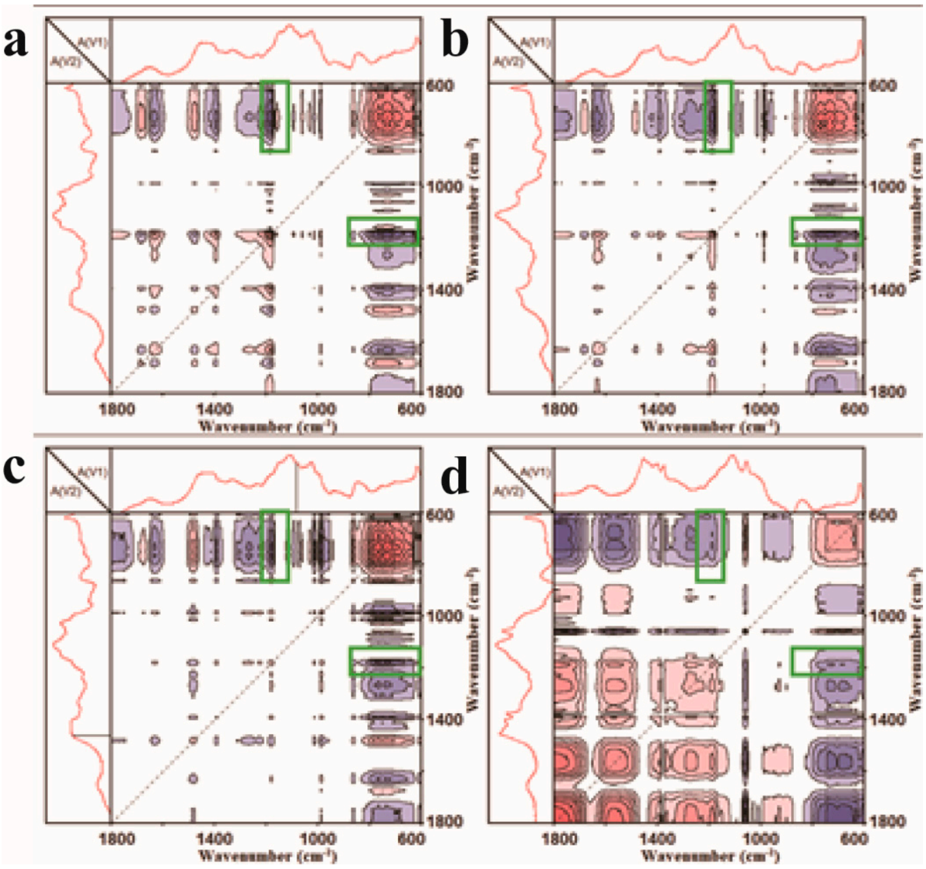

Figure S2. Synchronous FTIR spectra calculated from the temperature-dependent spectra of 25－77℃ in the range of 1800-600cm^{-1}. a) β-cyclodextrin-*cis*-jasmonone host-guest inclusion complex; b) β-cyclodextrin-cis-jasmonone blend; c) β-cyclodextrin-citronella host-guest inclusion complex; d) β-cyclodextrin-citronella blend. Pink and blue areas represent the positive and negative correlation intensity, respectively.

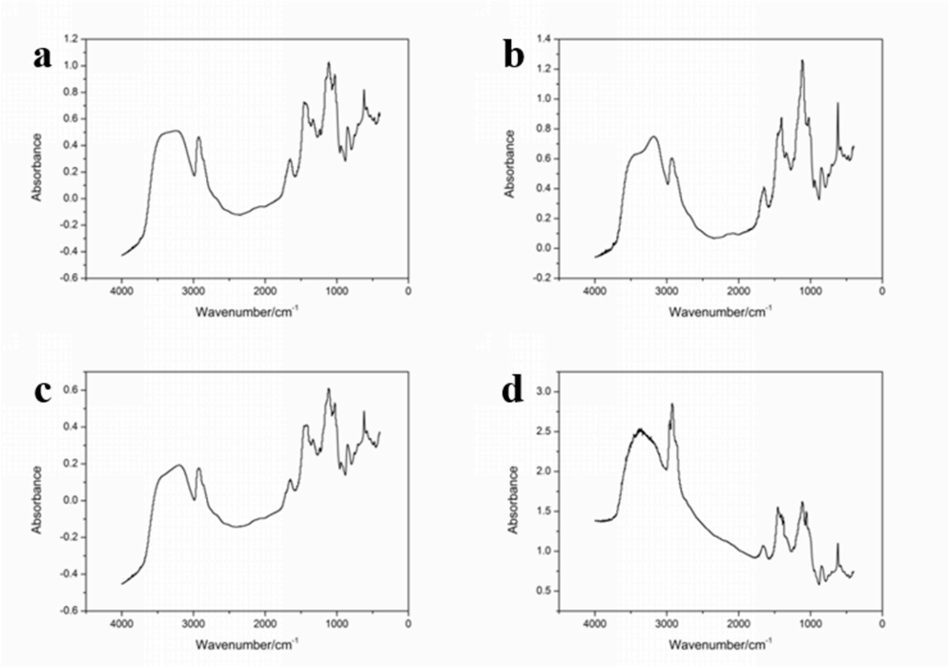

Figure S3. 1D-ATR-FTIR spectra of the a) *β*-cyclodextrin-*cis*-jasmonone host-guest inclusion complex; b) *β*-cyclodextrin- *cis*-jasmonone blend; c) *β*-cyclodextrin-citronella host-guest inclusion complex; d) *β*-cyclodextrin-citronella blend.

Table S1. Full width half maxima (FWHM) of the as-prepared and formalized fibers.

	Full width half maxima（FWHM）/cm^{-1}			
2θ/°	11.4°	19.4°	20.5°	22.5°
PVA	0.832	1.033	-	0.888
PVA/50CD	0.301	0.810	0.383	0.463
PVA/75CD	0.800	0.856	0.539	0.627
PVA/100CD	0.643	0.659	0.383	0.661
PVA/GA	0.798	1.015	-	1.209
PVA/50CD/GA	0.194	0.372	-	0.218
PVA/75CD/GA	1.065	0.892	-	0.854
PVA/100CD/GA	0.322	0.936	-	0.814

Table S2. Crystallite dimension of the as-prepared and formalized fibers.

	Crystallite dimension			
2θ/°	11.4°	19.4°	20.5°	22.5°
PVA	97	79	-	92
PVA/50CD	283	100	219	180
PVA/75CD	101	95	153	131
PVA/100CD	126	124	142	124
PVA/GA	101	80	-	67
PVA/50CD/GA	482	97	-	419
PVA/75CD/GA	76	91	-	96
PVA/100CD/GA	262	87	-	101

Table S3. *d*-spacing of the as-prepared fibers derived from XRD

d-spacing/Å	11.4°	19.4°	20.5°	22.5°
PVA	7.65	4.57	-	3.92
PVA/50CD	7.75	4.58	4.37	3.90
PVA/75CD	7.53	4.60	4.39	3.92
PVA/100CD	7.11	4.60	4.39	3.90
PVA/GA	7.72	4.60	-	3.83
PVA/50CD/GA	7.67	4.67	-	3.95
PVA/75CD/GA	7.54	4.68	-	3.90
PVA/100CD/GA	7.65	4.63	-	3.94

Table S4. The melting enthalpy and crystallization of PVA and PVA/CD fibers

	PVA	PVA/50CD	PVA/75CD	PVA/100CD
Melting Enthalpy $\triangle H_f$ /J/g	50.8	28.91	13.52	3.98
Crystallization	30.2%	17.2%	8.0%	2.4%

Table S5. The melting enthalpy and crystallization of PVA/GA and PVA/CD/GA fibers

	PVA/GA	PVA/50CD/GA	PVA/75CD/GA	PVA/100CD/GA
Melting Enthalpy $\triangle H_f$ /J/g	49.47	22.99	22.17	8.55
Crystallization	29.4%	13.7%	13.2%	5.1%

Table S6. Shrinkage of PVA/75CD crosslinked by different concentration of GA

Concentration g/L	10	15	20	25
Rate of shrinkage/%	36.8±2.3	28.7±3.1	19.5±2.6	/
